# Environmental Assessment and Blood Lead Levels of Children in Owino Uhuru and Bangladesh Settlements in Kenya

**DOI:** 10.5696/2156-9614-8.18.180605

**Published:** 2018-06-11

**Authors:** Nancy A. Etiang', Wences Arvelo, Tura Galgalo, Samwel Amwayi, Zeinab Gura, Jackson Kioko, Gamaliel Omondi, Shem Patta, Sara A. Lowther, Mary Jean Brown

**Affiliations:** 1 Ministry of Health, Kenya; 2 Jomo Kenyatta University of Agriculture and Technology, Kenya; 3 Division of Global Health Protection, Center for Global Health, Centers for Disease Control and Prevention, USA; 4 Division of Global Health Protection, Center for Global Health, Centers for Disease Control and Prevention, Kenya; 5 Mombasa County Department of Health Services, Kenya; 6 Harvard T.H. Chan School of Public Health

**Keywords:** childhood, blood, lead, environmental exposure, Kenya

## Abstract

**Background.:**

Lead exposure is linked to intellectual disability and anemia in children. The United States Centers for Disease Control and Prevention (CDC) recommends biomonitoring of blood lead levels (BLLs) in children with BLL ≥5 μg/dL and chelation therapy for those with BLL ≥45 μg/dL.

**Objectives.:**

This study aimed to determine blood and environmental lead levels and risk factors associated with elevated BLL among children from Owino Uhuru and Bangladesh settlements in Mombasa County, Kenya.

**Methods.:**

The present study is a population-based, cross-sectional study of children aged 12–59 months randomly selected from households in two neighboring settlements, Owino Uhuru, which has a lead smelter, and Bangladesh settlement (no smelter). Structured questionnaires were administered to parents and 1–3 ml venous blood drawn from each child was tested for lead using a LeadCare ^®^ II portable analyzer. Environmental samples collected from half of the sampled households were tested for lead using graphite furnace atomic absorption spectroscopy.

**Results:**

We enrolled 130 children, 65 from each settlement. Fifty-nine (45%) were males and the median age was 39 months (interquartile range (IQR): 30–52 months). BLLs ranged from 1 μg/dL to 31 μg/dL, with 45 (69%) children from Owino Uhuru and 18 (28%) children from Bangladesh settlement with BLLs >5 μg/dL. For Owino Uhuru, the geometric mean BLL in children was 7.4 μg/dL (geometric standard deviation (GSD); 1.9) compared to 3.7 μg/dL (GSD: 1.9) in Bangladesh settlement (p<0.05). The geometric mean lead concentration of soil samples from Owino Uhuru was 146.5 mg/Kg (GSD: 5.2) and 11.5 mg/Kg (GSD: 3.9) (p<0.001) in Bangladesh settlement. Children who resided <200 m from the lead smelter were more likely to have a BLL ≥5 μg/dL than children residing ≥200 m from the lead smelter (adjusted odds ratio (aOR): 33.6 (95% confidence interval (CI): 7.4–153.3). Males were also more likely than females to have a BLL ≥5 μg/dL (39, 62%) compared to a BLL<5 μg/dL [aOR: 2.4 (95% CI: 1.0–5.5)].

**Conclusions.:**

Children in Owino Uhuru had significantly higher BLLs compared with children in Bangladesh settlement. Interventions to diminish continued exposure to lead in the settlement should be undertaken. Continued monitoring of levels in children with detectable levels can evaluate whether interventions to reduce exposure are effective.

**Participant Consent.:**

Obtained

**Ethics Approval.:**

Scientific approval for the study was obtained from the Ministry of Health, lead poisoning technical working group. Since this investigation was considered a public health response of immediate concern, expedited ethical approval was obtained from the Kenya Medical Research Institute and further approval from the Mombasa County Department of Health Services. The investigation was considered a non-research public health response activity by the CDC.

**Competing Interests.:**

The authors declare no competing financial interests.

## Introduction

Lead exposure has been estimated to cause intellectual disability in approximately 600,000 children annually.^1^ In 2004, lead exposure was responsible for 143,000 deaths globally.^1^ There is no known safe threshold of blood lead in children since adverse effects have been shown to occur at a blood lead level (BLL) <10 μg/dL, which was previously thought to be safe.^2^ Lead exposure in young children may result in anemia, mental retardation, impaired intellectual and cognitive function, learning disabilities, poor attention, hearing disorders and decreased growth.^3^ Acute lead poisoning causes gastrointestinal disturbances (anorexia, nausea, vomiting, abdominal pain), hepatic and renal damage and neurological effects (malaise, drowsiness, encephalopathy), which may lead to convulsions, coma, and death.^4^ Lead exposure is often asymptomatic, and can remain undiagnosed and untreated. The United States Centers for Disease Control and Prevention (CDC) former Advisory Committee on Childhood Lead Poisoning Prevention recommended a reference value be used to identify children with elevated BLL; in 2012 this value was 5 μg/dL.^5^

Children are most often exposed to lead through ingestion of dust, soil, and water contaminated with lead. Children are at increased risk of lead poisoning compared to adults due to their physiological structure and behavior patterns.^6^ Lead is easily absorbed and distributed to blood and body organs through the developing gastrointestinal mucosa and blood brain barrier in children.^7^ Lead is poorly excreted, and most lead is sequestered in bone and slowly mobilized into the bloodstream. As a result, elevated blood lead levels (BLLs) take months to years to decrease, even in cases where external exposures have been reduced and chelation therapy has been instituted.^8^

During the last decade, large-scale measures have been implemented to reduce lead exposure globally. Dramatic reductions in BLLs have followed removal of lead from gasoline and paint products.^9^,^10^ Leaded paint used in houses built before 1950 remains the major source of lead exposure for American children.^11^ Children living in low-income countries are at increased risk of exposure to lead from multiple sources.^12^ Studies have described elevated BLLs in children living in communities involved in mining, smelting, and lead battery recycling.^13–15^ Additionally, outbreaks of lead intoxication have been associated with use of items containing lead such as water pipes, solders, and fittings, ceramic utensils, canned food, toys, traditional medicines, and cosmetics, such as *kohl*.^12^ Adults working in jobs or hobbies that generate lead dust carry the lead particles on clothes and shoes, and contribute to the lead dust in the child's home. These jobs include industries such as smelting, auto repair, firing ranges, painting, ceramics, pottery, electrical, wire and cable works, battery manufacturing and recycling, and stained glass.

In 2007, a lead battery recycling and smelting factory was established in Owino Uhuru settlement in Mombasa County along coastal Kenya. This provided job opportunities to the local members of the community. Investigations done in 2012 revealed high lead levels in soil, roof dust, wall dust and water. Prior to the investigation, blood samples from three children in Owino Uhuru were tested and found to have high blood lead levels of 23 μg/dl, 17 μg/dl, and 12 μg/dl. These findings prompted community members to seek compensation and immediate closure of the factory.^16^ The lead smelter was closed in July 2012. Despite this action, concerns remained regarding appropriate closure of the smelter and possible ongoing exposure from contaminated soil and dust. To enable public health authorities to make evidence-based actions to safeguard public health in the affected community, the Ministry of Health began an investigation on July 21, 2014. The investigation's objectives were to determine BLLs among children aged 12 to 59 months in the settlement, determine lead levels in the environment, and identify risk factors for elevated BLL in this population.

**Abbreviations**
*BLLs*: Blood lead levels
*CI*: Confidence interval
*GSD*: Geometric standard deviation
*OR*: Odds ratio

## Methods

**Figure 1:**
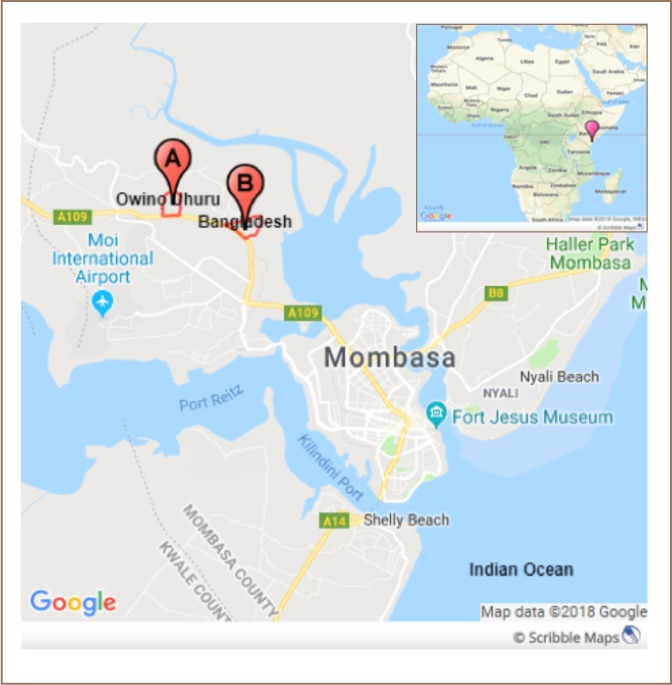
Map showing study sites of environmental assessment and blood lead levels of children in Mombasa County, Kenya

We conducted a population-based, cross-sectional study in two neighboring settlements in Mombasa County, located on the coastline of Kenya bordering the Indian Ocean *(Figure 1).* Owino Uhuru borders the northern side of the lead smelter and consists of about 500 households with a population of 2,500. The Bangladesh settlement, located on the southwestern side and more than 2 km upwind from the smelter, was included as the comparison site. Bangladesh settlement consists of roughly 5,000 households with a population of 25,000. Both settlements have similar housing characteristics and are located near the main Mombasa highway. Bangladesh settlement does not share water sources with Owino Uhuru and has no known battery recycling or smelting factories.

Village elders from the two settlements filled out a questionnaire with household membership details and ensured that all households with children 12–59 months of age were included in the sampling frame. We randomly selected 65 households from each settlement that had children aged 12–59 months using a table of random numbers. In each household, only one child aged 12–59 months was randomly selected to participate in the study. Since the lead smelter had not been in operation for one year, we excluded children who were less than one year old and those who had resided in either of the settlements for less than a year. Three attempts were made to return to houses that were vacant or where the parents refused to participate or were not available for interview. If a house was vacant after multiple attempts or the parent refused to provide consent, the neighboring household was visited. Survey teams were requested to track information using a form on household selection and response.

Data were collected using a pretested structured questionnaire translated to the local language, Kiswahili. We collected demographic information, including sex, age, clinical and treatment history, risk factors for elevated BLLs such as children's behavior, type of housing, sources of drinking or cooking water, the presence of a household member with known exposure to lead, and proximity of house to the lead battery recycling and smelting factory. Children's behavior habits related to the frequency of outdoor playing, washing hands before eating, and eating soil or sand. Global positioning system (GPS) coordinates of each participating house were taken at the entrance of the house, placed on a map, and used to calculate distances from the houses to the lead smelter. Distance of residence from smelter was categorized into two groups; less than 200 m and greater than or equal to 200 m. This distance was selected based on previous studies which indicated that children living near a point source, such as a lead smelter, were more likely to be lead-exposed compared to those further from the source.^17–19^

### Laboratory analysis

A venous blood specimen was collected from every child. The venipuncture site was thoroughly cleaned with alcohol wipes and wiped with dry gauze before the specimen was obtained. Approximately 1–3 ml of blood was collected in a vacutainer containing an anticoagulant, ethylenediaminetetraacetic acid (EDTA). In 10% of the children, a second venous specimen was obtained for quality control. We used certified lead-free blood collection supplies. All blood specimens were stored at 4°C and shipped on frozen packs to be analyzed at the Ministry of Health National Public Health Laboratory in Nairobi, Kenya. The blood specimens were tested for lead using LeadCare ^®^ II, a portable blood lead analyzer manufactured by Magellan Biosciences, Inc. (Chelmsford, MA, USA). Currently, the US Food and Drug Administration does not recommend testing of venous blood using LeadCare ^®^ II, however at the time of our investigation, the manufacturer's instructions stated that venous samples stored at 10° - 32°C could be analyzed within 24 hours of sample collection. The LeadCare^®^ II instrument quantifies BLLs from 3.3–65 μg/dL. These levels are measured with a level of detection accuracy of ± 3 μg/dL. For statistical analysis, levels below the detection limit were recorded as 3.3μg/dL. However, when a children's BLL was below the detection limit, parents and clinical health care providers were notified that the child's BLL was below the level of detection. Commercially prepared samples for high and low lead levels were used for quality control. Although 10% of the venous samples were obtained for further analysis, we could not analyze the samples, since the graphite furnace atomic absorption spectroscopy (GFAAS) at the government chemist in Nairobi was under repair.

### Environmental sampling

We randomly selected 50% of the houses where a child's blood specimen was collected to conduct environmental testing of dust, water and soil samples. The environmental samples were collected as per the US Housing and Urban Development protocol sampling procedures which is recognized by the US Environmental Protection Agency.^17^ We collected a composite soil sample from five areas of bare soil outside each house where the child usually plays using a fourounce plastic scoop and poured them in sealable plastic bags. Additional soil samples were obtained from the lead smelter compound and gate. We collected water used by the household for drinking in a 125 ml sample bottle. Dust was collected from the floor of the house by the multidirectional wipe sampling method using wet wipes over an area laid using a reusable template measuring 0.25 sq ft. The folded wipe was placed in a labelled sealable plastic bag. All the used materials were discarded in trash bags. To prevent contamination or exporting of lead dust, hands were cleaned with cleansing wipes after each sample collection and new gloves were worn before collecting samples in the next area. All environmental samples were placed separately in properly labeled clean, polythene bags. The environmental samples together with laboratory forms were sent to the Government Chemist Laboratory in Mombasa, Kenya. There they were analyzed for lead levels using GFAAS.

### Data management and analysis

Data were entered into a database using Epi InfoTM 7.1.4 (CDC, Atlanta, GA, USA). We compared the lead levels from the two settlements using a two-sample t-test after log transformation of the lead levels. We used a reference value of 5 μg/dL for elevated BLL.^5^ Environmental samples with elevated lead levels were defined as lead concentration in soil of >400 mg/Kg, >0.01 mg/L lead in drinking water, and lead loading in house dust of >40 μg/sq ft.^18–19^ The lead loading of house dust was obtained by multiplying micrograms of lead per gram of dust by the amount of dust on the surface measuring 0.25 sq ft. We also performed bivariate and multivariate analysis of factors associated with elevated BLLs. To determine the risk factors for BLL >5 μg/dL we designed a model for multivariate analysis and variables with p<0.2 in the bivariate analysis were added in the model. The variables included sex, being a resident of Owino Uhuru versus a resident of Bangladesh settlement, distance less than 200 m from factory compared to 200 m or greater, and spending >6 hours per day in compound and playing outside.

### Ethics approval

Scientific approval for the study was obtained from the Ministry of Health, lead poisoning technical working group. Since this investigation was considered a public health response of immediate concern, expedited ethical approval was obtained from the Kenya Medical Research Institute and further approval from the Mombasa County Department of Health Services. The investigation was considered a non-research public health response activity by the CDC. Informed consent was gathered from parents before enrollment.

## Results

We enrolled 130 children aged 12–59 months, 65 from each settlement. In the 41 (31.5%) households with two or more children aged 12–59 months, one child was randomly selected. Survey teams failed to use the household enrollment tracking forms, therefore it was not possible to calculate nonresponse. However, of those households ultimately selected, none of the families refused to participate. Median age was 39 months (interquartile range (IQR): 30–52), and 59 (45%) were males. Of the 65 children from Owino Uhuru, 31 (48%) resided within 200 m of the lead smelter and 16 (25%) lived in a household with at least one adult working in any job involving lead within the last five years. Nine (14%) out of the 65 children from Bangladesh settlement lived in a household with at least one adult working in any job involving lead for last five years *(Table 1)*.

**Table 1:** Characteristics of Children Investigated for Blood Lead in Owino Uhuru and Bangladesh Settlements, Mombasa County, Kenya, January 2015

**Characteristics**	**Owino Uhuru (N = 65) n (%)**	**Bangladesh (N = 65) n (%)**	**Total (N = 130) n (%)**
Male	13 (52)	58 (55)	71 (55)
Median age in months (IQR)	42 (31 – 50)	36 (27 – 52)	39 (30 – 52)
Age Groups (Months)			
12 – 23	10 (15)	14 (22)	24 (19)
24 – 35	15 (23)	18 (28)	33 (25)
36 – 47	18 (28)	6 (9)	24 (19)
48 – 59	22 (34)	27 (42)	49 (38)
Residence < 200 m from lead smelter	31 (48)	0 (0)	31 (24)
Living in house with adults working in job involving lead for last 5 years	16 (25)	9 (14)	25 (19)
Blood Lead Levels (μg/dL)			
0.7 – 4.9	20 (31)	47 (72)	67 (52)
5 – 9.9	25 (39)	13 (20)	38 (29)
10 – 19.9	16 (25)	5 (8)	21 (16)
20 – 30.5	4 (6)	0 (0)	4 (3)
Lead content of environmental samples			
Soil^*^ (>400 mg/Kg)	7 (21)	1 (3)	8 (12)
House dust^**^ (>40 μg/sq ft)	4 (13)	3 (9)	7 (11)
Drinking water^*^ (>0.01 mg/L)	1 (3)	14 (44)	15 (24)

Abbreviation: IQR, interquartile range

^*^ n = 66

^**^n = 65

BLLs among sampled children ranged from 1 μg/dL to 31 μg/dL. Overall, 63 (48%) children had BLLs of >5 μg/dL and 20 (19%) children had BLLs of >10 μg/dL. There were 25 (39%) children from Owino Uhuru and 13 (20%) children from Bangladesh settlement with BLLs of 5 – 9.9 μg/dL. Twenty (31%) children from Owino Uhuru and five (8%) children from Bangladesh settlement had BLLs of 10–19.9 μg/dL. Four (6%) children in Owino had BLLs of 20 – 31 μg/dL. For Owino Uhuru, the geometric mean BLLs in children were 7.4 μg/dL (geometric standard deviation (GSD): 1.9) compared to 3.7 μg/dL (GSD: 1.9) for Bangladesh settlement (p<0.05).

Of the 33 households in Owino Uhuru selected for environmental assessment, house dust and soil were collected from 33 (100%) and drinking water from 30 (91%) of households.

In Bangladesh settlement, house dust was collected from 34 (97%), soil from 33 (94%) and water from 35 (100%) of the 35 households selected. The soil from Owino Uhuru had lead concentrations ranging from 0.4 to 2,655 mg/Kg, whereas in Bangladesh settlement, soil lead ranged from 0.1 to 653 mg/Kg. There were seven (21%) soil samples from Owino Uhuru and one (3%) from Bangladesh settlement with soil lead concentration ≥400 mg/Kg. The geometric mean lead concentration of soil samples from Owino Uhuru details was higher than the geometric mean lead concentration of soil samples from Bangladesh settlement (11.5 mg/Kg; GSD: 3.9) (p<0.001). Additional soil samples obtained in the factory compound had the highest lead concentration of 26,837 mg/Kg, followed by the one collected at the factory gate of 2,381 mg/Kg. The geometric mean lead loading (1.5 μg/sq ft; GSD: 12.3) of house dust from Owino Uhuru was higher than the geometric mean lead loading (0.3 μg/sq ft; GSD: 6.4) of house dust samples from Bangladesh settlement (p<0.01). The geometric mean lead concentration (0.003 mg/L; GSD: 10.0) of water samples from Owino Uhuru was lower than the geometric mean lead concentration (0.01 mg/L; GSD: 6.1) of water samples from Bangladesh settlement (p<0.05).

The bivariate analysis found that children who resided <200 m from the factory were more likely to have BLLs ≥5 μg/dL (odds ratio (OR): 30.0, 95% confidence interval (CI): 6.6–131.2) than those who resided ≥200 m Refrom the battery factory *(Table 2).* Children living in homes with soil lead concentrations of ≥100 mg/Kg had a higher risk of having a BLL ≥5 μg/dL (OR: 3.1, 95% CI: 1.1–8.4). Children who spent their day in the compound were at higher risk of BLLs ≥5 μg/dL than those who spend most of their day in school (OR: 2.7, 95% CI: 1.1–6.9). Increased BLL was not associated with children living in households with adults whose occupation or hobbies for the last five years had exposed them to lead. The main source of drinking or cooking water in all the households was obtained from tanks with water supplied by the local municipality. Children from houses that used other sources of water such as wells (15; 24%), rain water (5; 8%), and boreholes (12; 19%) did not show elevated BLLs *(Table 2).*Variables with p<0.2 in the bivariate analysis were included in the multivariate model. These included sex of the child, being a resident of Owino Uhuru versus a resident of Bangladesh settlement, residing less than 200 m from the factory compared to 200 m or greater, and spending >6 hours in the compound and playing outside. Children residing <200 m from the lead smelter had a higher risk of BLL ≥5 μg/dL compared to residing 200 m or farther from the lead smelter (adjusted odds ratio (aOR): 33.6, 95% CI: 7.4–153.3), as well as male children (39; 62%) compared to female children (aOR: 2.4, 95% CI: 1.0–5.5).

**Table 2:** Bivariate Analysis of Factors Associated with Blood Lead Levels Among Children in Owino Uhuru and Bangladesh Settlements, Mombasa County, Kenya, January 2015

**Variable**	**Blood Lead Level**		**Odds Ratio**
	**≥5 μg/dL (n=63) n (%)**	**< 5 μg/dL (n=67) n (%)**	**(95% Confidence Interval)**
Sex			
Female	24 (38)	35 (52)	Ref
Male	39 (62)	32 (48)	1.8 (0.9–3.6)
Age Groups (Months)			
12 – 23	9 (14)	15 (22)	Ref
24 – 35	16 (25)	17 (25)	1.6 (0.5 – 4.5)
36 – 47	12 (19)	12 (18)	1.6 (0.5 – 5.2)
48 – 59	26 (41)	23 (34)	1.9 (0.6 – 5.1)
Settlement			
Bangladesh	18 (29)	47 (70)	Ref
Owino Uhuru	45 (71)	20 (30)	5.9 (2.8 – 12.5)
Residence from lead smelter			
>200 m	33 (52)	65 (97)	Ref
<200 m	30 (48)	2 (3)	30 (6.6 – 131.2)
Family member reported occupationally exposed to lead			
No	50 (79)	55 (82)	Ref
Yes	13 (21)	12 (18)	1.2 (0.5 – 2.9)
Primary drinking/cooking water source – municipal	63 (100)	67 (100)	1.0 (CI undefined)
Other sources of drinking/cooking water			
Rain Water	5 (8)	10 (15)	Ref
Well	15 (24)	11 (16)	2.7 (0.7 – 10.3)
Bore hole	12 (19)	9 (13)	2.7 (0.7 – 10.6)
Floor Type – Mud			
No	45 (71)	45 (77)	Ref
Yes	18 (29)	22 (33)	0.8 (0.4 – 1.7)
Floor Type – Concrete			
No	20 (32)	25 (37)	Ref
Yes	43 (68)	42 (63)	1.3 (0.6 – 2.6)
Consumes vegetables/fruits from own garden/neighbor who grows			
No	49 (78)	54 (81)	Ref
Yes	14 (22)	13 (19)	1.2 (0.5 – 2.8)
Spends most of day			
In school	16 (25)	9 (13)	Ref
Inside compound	33 (52)	51 (76)	0.4 (0.1 – 0.9)
In the house	6 (10)	5 (7)	0.4 (0.1 – 0.9)
Somewhere else in the village	8 (13)	2 (3)	2.6 (0.4 – 13.0)
Eat Soil or Sand			
No	26 (59)	31 (46)	Ref
Yes	37 (41)	36 (54)	1.2 (0.6 – 2.5)
Doesn't wash hands before eating			
No	43 (68)	50 (75)	Ref
Yes	20 (32)	17 (25)	1.4 (0.6 – 2.9)
Plays outside ≥ 6 hours			
No	23 (36)	16 (24)	Ref
Yes	40 (64)	51 (76)	0.5 (0.3 – 1.2)
Wall of dwelling – mud			
No	18 (29)	21 (31)	Ref
Yes	45 (71)	46 (69)	1.1 (0.5 – 2.4)
Soil lead (mg/Kg)			
<100	13 (39)	22 (67)	Ref
≥100	20 (61)	11(33)	3.1 (1.1 – 8.4)
<400	28 (85)	30 (91)	Ref
≥400	5 (15)	3 (9)	1.8 (0.4 – 8.1)
House dust lead			
<40 μg/sq ft	30 (94)	28 (85)	Ref
≥40 μg/sq ft	2 (6)	5 (15)	0.4 (0.1 – 2.1)
Drinking water lead			
<0.01 mg/L	28 (87)	23 (67)	Ref
≥0.01 mg/L	4 (13)	11 (33)	0.3 (0.1 – 1.0)

## Discussion

Findings from our study demonstrated elevated BLLs among children aged 12–59 months in Owino Uhuru in comparison to similar children from Bangladesh settlement. Elevated lead levels were also found in the soil in Owino Uhuru. Close proximity of houses to the lead smelter and male sex were identified as risk factors for elevated BLLs. Our findings support the hypothesis that BLLs among children in Owino Uhuru are different from the BLLs of children from Bangladesh settlement and the difference is associated with proximity to the lead smelter. Further interventions are required to reduce lead exposure in the affected community.

Recent studies have demonstrated significant neurological deficits in children with detectable BLLs.^21^ Our study found that two-thirds of the tested children in Owino Uhuru and a third of the children from Bangladesh settlement had BLLs above 5 μg/dL. Therefore, children with elevated BLLs in this community need to be followed up and take actions to reduce lead levels and children's further exposure to lead. None of the children had BLLs ≥45 μg/dL, and therefore did not require chelation therapy.^22^ Although we did not test for cognitive and behavioral problems related to lead poisoning, the clinical symptoms, such as abdominal pains reported by some of the children were non-specific to lead poisoning.^24^

Children from Owino Uhuru had higher BLLs than children in Bangladesh settlement. Owino Uhuru settlement has similar housing and environmental characteristics to Bangladesh settlement; however, Bangladesh settlement has never had a battery factory. Elevated BLLs in children have been similarly documented in studies of children exposed to industrial emissions from battery recycling and smelting activities. In these studies, 59% of children in Jamaica, 80% of children in Nicaragua and 91% of children from Haina, Dominican Republic were found to have a BLL >10 μg/dL.^8^,^14^,^23^ It is likely that the difference in BLL is associated with the lead smelter located in Owino Uhuru settlement. There were significant elevations in soil lead concentration in Owino Uhuru. A study carried out during the same period near used lead acid battery recycling operations in Nairobi slums, Kenya, also showed elevated levels of lead in soil and dust samples. The researchers predicted that almost all of the children living in the slums would have BLLs above 34 μg/dL.^24^ A review of nine studies measuring blood lead levels and point source exposures suggests that soil less than 270 meters from a point source, such as a lead smelter, is more likely to be heavily contaminated compared to soil further distant from the source.^25–33^

The finding that a third of the children from Bangladesh settlement had a BLL ≥ 5 μg/dL suggests that there could be other sources of lead in Bangladesh settlement, and that in Owino Uhuru, BLLs may not only be associated with lead smelting activities. Water lead levels were below action level in both communities, however a slightly higher lead concentration was observed in water samples from Bangladesh settlement. This could be a possible reason for the elevated BLLs among some children in Bangladesh settlement. Other sources of lead need to be identified.

Our study has some limitations. First, study teams did not use the household tracking form to document the random selection of households and from which nonresponse could have been calculated. Therefore, enrolled children may not truly represent a random selection. Our results are suggestive of oversampling of the 48–59-month-old group, who had higher BLLs. That might be because caregivers were concerned about the effects of the factory among the children of this age who were living in the community when the factory was operational, and therefore more likely to have enrolled older children into the study. Alternatively, households with younger children might have avoided enrollment by not being present when study teams visited, out of reticence to have blood drawn on younger children. Second, the study sample size focused on determining differences in BLLs in the two settlements. We did not have enough statistical power to study other potential risk factors between the children with different BLLs. Nonetheless, despite these limitations, it appears that childhood lead exposure from the environment persists. Younger children aged 12–23 months who were born after the closure of the factory also had elevated BLLs, which indicates that their exposure was from residual lead in the environment.

## Conclusions

There were significantly high BLLs among children in Owino Uhuru associated with high soil lead concentrations and proximity to the lead smelter. The most immediate priority is reduction of blood lead and remediation of the contaminated soil, particularly near the lead smelter.

Following this study, in 2015, the Mombasa county government established a medical camp at Owino Uhuru where community members were treated for various ailments and children were provided with nutritional supplements and hematinics. Parents and caregivers were advised to perform activities to minimize further absorption of lead. These include providing children with a well-balanced diet, washing children's hands and toys regularly, ensuring cleanliness of the house by preventing entering the house with shoes and regularly wet mopping the floor and other areas in the house. A national task force led by the National Environmental and Management Authority was formed to undertake actions to remediate the soil in Owino Uhuru. The taskforce identified a nongovernmental organization that would carry out the remediation. Community members have shown a positive attitude about the actions undertaken and this highlights the need for continuous community involvement.

Given that this study was carried out three years ago, retesting of capillary blood lead levels using a LeadCare ^®^ II analyzer would help determine whether there is continued exposure and identify children with elevated BLLs. Children with elevated BLLs will require regular follow-up testing to monitor BLL, hemoglobin and assessment of cognitive behavior. Health care workers need to identify children with poor nutritional status and provide them with the necessary dietary supplements.

The high lead level of soil in Owino Uhuru illustrates the need for coordinated efforts to remediate the environment. This requires removal of the first several inches of soil and replacing with clean fill, which may necessitate the removal of children from their homes until the environment is confirmed to be safe. The factory should remain closed and children's homes regularly evaluated to prevent further lead exposure. These efforts may require collaboration and coordination from the government, non-governmental organizations and the affected communities. Further studies need to be carried out to determine whether the factory is an independent risk factor for lead exposure among children in this community. This study highlights the importance of carrying out environmental assessments and screening of children in other areas of possible lead contamination and undertaking interventions to minimize the risk and consequences of exposure to lead.
